# Progress in implementation of WHO FCTC Article 14 and its guidelines: a survey of tobacco dependence treatment provision in 142 countries

**DOI:** 10.1111/add.13903

**Published:** 2017-08-02

**Authors:** Kapka Nilan, Martin Raw, Tricia M. McKeever, Rachael L. Murray, Ann McNeill

**Affiliations:** ^1^ UK Centre for Tobacco and Alcohol Studies, School of Medicine University of Nottingham Nottingham UK; ^2^ UK Centre for Tobacco and Alcohol Studies, Institute of Psychiatry Psychology and Neuroscience (IoPPN) King's College London London UK

**Keywords:** Article 14 guidelines, FCTC Article 14, middle‐ and low‐income countries, tobacco cessation, tobacco control, tobacco dependence treatment

## Abstract

**Aims:**

To (1) estimate the number of Parties to the Framework Convention on Tobacco Control (FCTC) providing tobacco dependence treatment in accordance with the recommendations of Article 14 and its guidelines; (2) assess association between provision and countries’ income level; and (3) assess progress over time.

**Design:**

Cross‐sectional study.

**Setting:**

Online survey from December 2014 to July 2015.

**Participants:**

Contacts in 172 countries were surveyed, representing 169 of the 180 FCTC Parties at the time of the survey.

**Measurements:**

A 26‐item questionnaire based on the Article 14 recommendations including tobacco treatment infrastructure and cessation support systems. Progress over time was assessed for those countries that also participated in our 2012 survey and did not change country income level classification.

**Findings:**

We received responses from contacts in 142 countries, an 83% response rate. Overall, 54% of respondents reported that their country had an officially identified person responsible for tobacco dependence treatment, 32% an official national treatment strategy, 40% official national treatment guidelines, 25% a clearly identified budget for treatment, 17% text messaging, 23% free national quitlines and 26% specialized treatment services. Most measures were associated positively and significantly with countries’ income level (*P* < 0.001). Measures not associated significantly with income level included mandatory recording of tobacco use (30% of countries), offering help to health‐care workers (HCW) to stop using tobacco (44%), brief advice integrated into existing services (44%), and training HCW to give brief advice (81%). Reporting having an officially identified person responsible for tobacco cessation was the only measure with a statistically significant improvement over time (*P* = 0.0351).

**Conclusion:**

Fewer than half of countries that are Parties to the Framework Convention on Tobacco Control have implemented the recommendations of Article 14 and its guidelines, and for most measures, provision was greater the higher the country's income. There was little improvement in treatment provision between 2012 and 2015 in all countries.

## Introduction

Article 14 of the World Health Organization (WHO) Framework Convention on Tobacco Control (FCTC) requires Parties to promote tobacco cessation and implement effective measures to help tobacco users quit 1. The Article 14 guidelines, adopted at the fourth Conference of the Parties in 2010 2 provide further details on the implementation of Article 14 3, including the key elements of basic treatment infrastructure and support systems that Parties should implement, and were outlined in our previous paper 4.

Our previous survey found that most countries, especially middle‐ and low‐income countries, had not fully implemented the Article 14 guidelines recommendations 4. The aims of this study were to (1) estimate the current level of provision of tobacco dependence treatment; (2) assess association between provision and countries’ income level; and (3) assess progress since the previous survey.

## Methods

We identified contacts in 169 FCTC Parties at the time of the survey. We excluded the EU as a non‐state Party and were unable to find contacts for 10 Parties. Our survey sample consisted of 168 Parties and the United Kingdom (which is one Party but made up of four countries (England, Northern Ireland, Scotland and Wales), each with a different health‐care system, which were therefore surveyed individually)—172 countries in all.

We approached the contacts from our previous survey by e‐mail in December 2014, inviting them to take part in this survey. For contacts we could not reach or who did not reply, we identified new ones with the help of the WHO regional offices, the Framework Convention Alliance (FCA), Action on Smoking and Health (ASH) in the United States, the InterAmerican Heart Foundation and other professional networks and contacts. The contacts included tobacco treatment specialists and government and non‐government representatives, involved in tobacco cessation or tobacco control in their countries.

The questionnaire was based on our previous one, with a few questions improved for clarity or for better alignment with the Article 14 guidelines. However, this time we conducted two surveys: one on treatment provision and one on treatment guidelines. In this paper we report the findings of the treatment provision survey only. The guidelines results are reported in a second paper. The questionnaire was translated into French, Spanish and Russian and sent electronically as a Word attachment. A link to an online version of the questionnaire in English was also provided. The treatment questionnaire (see Appendix S1) covered basic treatment infrastructure (an officially identified person in government responsible for cessation, a national treatment strategy, a treatment budget, treatment guidelines, mandatory recording of tobacco use in medical notes, training of health‐care workers (HCW) to give brief advice and help to stop using tobacco) and cessation support systems (brief advice integrated into existing services, medications, mass media campaigns, quitlines, text messaging, specialized treatment services). In order to help validate the responses, we asked the respondents to send supporting information and references with their answers where possible. We sent reminders every 2 weeks until July 2015, when the survey was closed.

Survey data were analysed by country World Bank (WB) income level on 1 July 2014 5. The statistical analyses were conducted in Stata version 14. We used the χ^2^ statistic to test association between implementation of measures and countries’ income level. We used the McNemar exact test to assess changes in implementation between our two surveys.

## Results

### Survey response rate

We received responses from contacts in 142 countries, an 83% response rate. Response rates by income level were: 92% in high‐income countries (HIC, *n* = 49), 80% in upper middle‐income countries (UMIC, *n* = 40), 82% in lower middle‐income countries (LMIC, *n* = 36) and 68% in low‐income countries (LIC, *n* = 17).

### Basic treatment infrastructure

According to respondents, just more than half of countries had a government official responsible for treatment (54%). Only a quarter reported having a clearly identified treatment budget (25%), fewer than a third an official treatment strategy (32%) and fewer than half official national treatment guidelines (40%). Just under a third reported mandatory recording of tobacco use in medical notes (30%) and fewer than half that cessation support was offered to HCW to stop using tobacco (44%) (Table 1). Just more than 80% reported training some HCW to give brief advice and more than a third incorporated tobacco cessation into training curricula of health‐care students and workers (36%). There was a significant difference in provision across income level for treatment budget, official treatment strategy, national treatment guidelines and cessation in training curricula, but not for the other measures (Table 1).

**Table 1:** Tobacco treatment infrastructure by World Bank income level.

Question, % Yes (n)	All (142)	HIC (49)	UMIC (40)	LMIC (36)	LIC (17)	P‐value for difference across income level
Is there an officially identified person in government (or contracted by government) who is responsible for tobacco dependence treatment?	**54** (76)	**57** (28)	**63** (25)	**53** (19)	**24** (4)	0.052
Does your country have a clearly identified budget for tobacco dependence treatment?	**25** (35)	**43** (21)	**20** (8)	**17** (6)	**0** (0)	0.001
Does your country have an official, written, national tobacco treatment strategy?	**32** (46)	**51** (25)	**25** (10)	**31** (11)	**0** (0)	< 0.001
Does your country have official national tobacco treatment guidelines?	**40** (57)	**63** (31)	**38** (15)	**28** (10)	**6** (1)	< 0.001
Is it mandatory to record patients’ tobacco use in medical notes in your country?	**30** (42)	**39** (19)	**25** (10)	**25** (9)	**24** (4)	0.425
Does your country offer help to health‐care workers and other relevant groups to stop using tobacco?	**44** (63)	**53** (26)	**53** (21)	**25** (9)	**41** (7)	0.132
Are health‐care workers trained to give brief advice? (Yes, some)	**81** (100)	**73** (36)	**80** (32)	**58** (21)	**65** (11)	0.129
Is tobacco cessation incorporated into the training curricula of health‐care students and workers?	**36** (51)	**59** (29)	**28** (11)	**19** (7)	**24** (4)	< 0.001

HIC = high‐income countries; UMIC = upper middle‐income countries; LMIC = lower middle‐income countries; LIC = low‐income countries. Bold data indicate percentages.

### Cessation support systems

#### Mass media campaigns

Overall, only 13% of respondents reported mass media campaigns promoting cessation in the past 6 months: 23% of HIC, 15% of UMIC, 3% of LMIC and 6% of LIC (*P* = 0.002 for difference across income level, Table 2).

**Table 2:** Tobacco cessation support systems by World Bank income level.

Question, % Yes (n)	All (142)	HIC (49)	UMIC (40)	LMIC (36)	LIC (17)	P‐value for difference across income level
Mass media campaigns promoting cessation in the past 6 months?	**13** (19)	**23** (11)	**15** (6)	**3** (1)	**6** (1)	0.002
Brief advice integrated into existing services?	**44** (62)	**51** (25)	**48** (19)	**31** (11)	**41** (7)	0.281
Cessation support via text messaging?	**17** (24)	**35** (17)	**8** (3)	**11** (4)	**0** (0)	0.001
Quitlines						
A free national quitline in all major regions?	**23** (33)	**53** (26)	**15** (6)	**3** (1)	**0** (0)	< 0.001
A free quitline but only in selected regions?	**6** (9)	**4** (2)	**10** (4)	**8** (3)	**0** (0)	
A free national quitline but not tobacco only?	**8** (11)	**10** (5)	**8** (3)	**6** (2)	**6** (1)	
People answering always or almost always?	**82** (42)	**87** (27)	**85** (11)	**50** (3)	**100** (1)	
Offer multiple sessions with counsellors calling back to offer support?	**51** (26)	**66** (20)	**39** (5)	**17** (1)	**0** (0)	
Refer callers to local specialist treatment services?	**84** (43)	**87** (27)	**92** (12)	**50** (3)	**100** (1)	
Offer information about tobacco cessation medications?	**67** (34)	**74** (23)	**62** (8)	**33** (2)	**100** (1)	
Specialized tobacco dependence treatment service						
A network of support covering the whole country?	**26** (37)	**55** (27)	**20** (8)	**6** (2)	**0** (0)	< 0.001
Treatment support in selected areas?	**37** (53)	**35** (17)	**45** (18)	**33** (12)	**35** (6)	
Free to users?	**48** (43)	**55** (24)	**54** (14)	**14** (2)	**50** (3)	
Partially free to users?	**38** (35)	**36** (16)	**31** (8)	**57** (8)	**50** (3)	
Not free to users?	**12** (11)	**7** (3)	**15** (4)	**29** (4)	**0** (0)	

HIC = high‐income countries; UMIC = upper middle‐income countries; LMIC = lower middle‐income countries; LIC = low‐income countries. Bold data indicate percentages.

#### Brief advice

According to respondents, brief advice was integrated into existing services in fewer than half of countries (44%): 51% of HIC, 48% of UMIC, 31% of LMIC and 41% of LIC (*P* = 0.281 for difference across income level, Table 2).

#### Text messaging

Overall, 17% of respondents reported that text messaging support was available in their countries: 35% of HIC, 8% of UMIC, 11% of LMIC and no LIC (*P* < 0.001 for difference across income level, Table 2).

#### Quitlines

Free quitlines with national coverage were reported by 23% of respondents overall: 53% of HIC, 15% of UMIC, 3% of LMIC and no LIC (*P* < 0.001 for difference across income level, Table 2). Quitlines in selected regions, or helplines that were not tobacco‐only, were reported in fewer than 10% of countries.

More than 80% of respondents reported that all quitlines had people answering always or almost always, and referred callers to local treatment services. Just more than half offered multiple sessions with counsellors calling back, and 67% provided information about tobacco cessation medications (Table 2).

#### Specialized treatment support

Respondents in just more than a quarter of countries (26%) reported a network of specialized tobacco dependence treatment services covering the whole country: 55% of HIC, 20% of UMIC, 6% of LMIC and no LIC (*P* < 0.001 for difference across income level, Table 2).

An additional third (37%) reported specialized treatment support in selected areas: 35% of HIC, 45% of UMIC, 33% of LMIC and 35% of LIC.

Specialized treatment support was free to users in 48% of countries that offered it: 55% of HIC, 54% of UMIC, 14% of LMIC and 50% of LIC (Table 2).

#### Access to cessation support

**Figure 1:**
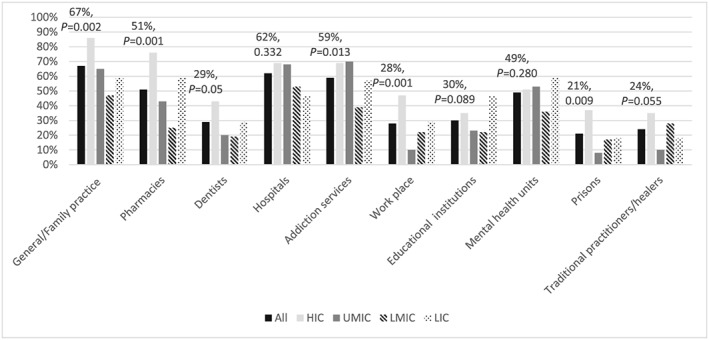
Access to cessation support in different settings. ‘Can tobacco users get help to stop smoking in the following settings?’. The bars show ‘Yes’ responses overall (black) then by income‐level in the order high‐income countries (HIC); upper middle‐income countries (UMIC); lower middle‐income countries (LMIC); low‐income countries (LIC). P‐values are for difference across income level

Respondents in more than half of countries reported that tobacco users could access cessation support from general practitioners (GPs) (67%), hospitals (62%), addiction services (59%) and pharmacies (51%) and in 49% of mental health settings. Cessation support from dentists, educational institutions, work‐places and prisons could be accessed in fewer than a third of countries. Access to cessation support from GPs, pharmacies, dentists, addiction services, work‐places and prisons was significantly less in middle‐ and low‐income countries than in high‐income countries (Fig. 1).

#### Availability of cessation medications

In approximately three‐quarters of countries, respondents reported that nicotine patches and gum were available, ranging from 98% of HIC to 47% of LIC (*P* < 0.001 for difference across income level). Availability of bupropion and varenicline also decreased with income level. Bupropion was reported to be available in 90% of HIC and 18% of LIC (*P* < 0.001 for difference across income level), varenicline in 88% of HIC and 6% of LIC (*P* < 0.001 for difference across income level, (Table 3). Cytisine was reported to be available only in 14% of countries overall (*P* = 0.692 for difference across income level).

**Table 3:** Availability of cessation medications by World Bank income level.

Medication	Available, % Yes (n)					P‐value for difference across income level
	All (142)	HIC 49)	UMIC (40)	LMIC (36)	LIC (17)	
NRT patch	**74** (104)	**98** (48)	**73** (29)	**53** (19)	**47** (8)	<0.001
NRT gum	**72** (102)	**96** (47)	**60** (24)	**61** (22)	**53** (9)	< 0.001
Other NRT	**45** (63)	**69** (34)	**40** (16)	**25** (9)	**24** (4)	< 0.001
Bupropion	**60** (84)	**90** (44)	**58** (23)	**39** (14)	**18** (3)	< 0.001
Varenicline	**54** (76)	**88** (43)	**48** (19)	**36** (13)	**6** (1)	< 0.001
Nortriptyline	**33** (46)	**53** (26)	**28** (11)	**19** (7)	**12** (2)	0.001
Cytisine	**14** (19)	**10** (5)	**13** (5)	**19** (7)	**12** (2)	0.692

HIC = high‐income countries; UMIC = upper middle‐income countries; LMIC = lower middle‐income countries; LIC = low‐income countries; NRT = nicotine replacement therapy.

#### Affordability of cessation medications

In countries where cessation medications were available, most respondents rated them as affordable, but with a clear gradient from high‐ to low‐income countries. For example, nicotine gum was rated affordable in 85% of HIC, 58% of UMIC, 45 of LMIC and 33% of LIC (*P* < 0.001 for difference across income level); varenicline was rated affordable in fewer than a third of UMIC and LMIC and in no LIC. There was no income gradient in the affordability of cytisine (*P* = 0.821) (Table 4).

**Table 4:** Affordability of cessation medications by World Bank income level.

Medication	Affordable, % Yes (n)					P‐value for difference across income level
	All	HIC	UMIC	LMIC	LIC	
NRT patch	**63** (65)	**79** (36)	**52** (15)	**53** (10)	**25** (2)	< 0.001
NRT gum	**66** (67)	**85** (40)	**58** (14)	**45** (10)	**33** (3)	< 0.001
Other NRT	**68** (43)	**94** (32)	**38** (6)	**44** (4)	**25** (1)	< 0.001
Bupropion	**57** (48)	**73** (32)	**43** (10)	**36** (5)	**33** (1)	< 0.001
Varenicline	**54** (41)	**77** (33)	**32** (6)	**15** (2)	**0** (0)	< 0.001
Nortriptyline	**59** (27)	**65** (17)	**55** (6)	**57** (4)	**0** (0)	0.003
Cytisine	**68** (13)	**80** (4)	**80** (4)	**57** (4)	**50** (1)	0.821

The percentages are calculated by dividing the number of countries where the medication is affordable by the number of countries where the medication is available (*n*), as shown in Table 3. HIC = high‐income countries; UMIC = upper middle‐income countries; LMIC = lower middle‐income countries; LIC = low‐income countries; NRT = nicotine replacement therapy.

#### Changes in treatment provision since 2012

We compared our current results with our previous survey in 2012, for those measures which were comparable, for a subset of 99 countries that completed both surveys and did not change their WB income level: 36 HIC, 28 UMIC, 23 LMIC and 12 LIC (Table 5).

**Table 5:** Country respondents in 2012 and 2015 by income.

Countries WB income category	Responded in 2012 survey, n	Responded in 2015 survey, n	Excluded from longitudinal analyses (did not respond or changed income category), n	Included in longitudinal analyses, n
Low‐income	19	17	5	12
Lower middle‐income	30	36	13	23
Upper middle‐income	36	40	12	28
High‐income	36	49	13	36
All	121	142	43^a^	99

a The 43 excluded countries included 23 that did not respond in the 2012 survey, 12 that did not respond in the 2015 survey and 10 that changed their World Bank (WB) income category.

Overall, while provision of some measures reportedly increased over time, such as having a government official responsible for cessation, an increase of 13 percentage points (*P* = 0.0351) and treatment guidelines, an increase of 9 percentage points (*P* = 0.629), there was little change in others, such as mass media campaigns promoting cessation, mandatory recording of tobacco use in medical notes, an official treatment budget and having a free national quitline (Table 6).

**Table 6:** Changes in treatment provision from 2012 to 2015.

Question	2012 % Yes (n)	2015 % Yes (n)	Change	P‐ value for change
Government official responsible for cessation?	**40** (40)	**54** (53)	13	0.035
Treatment budget?	**22** (22)	**23** (23)	1	1.00
Treatment guidelines?	**46** (46)	**56** (55)	9	0.629
Mandatory recording tobacco use in patients’ notes?	**21** (21)	**26** (26)	5	0.774
Health‐care workers offered help to stop using tobacco?	**46** (46)	**42** (42)	–4	0.487
Mass media campaigns promoting cessation?	**55** (54)	**56** (55)	1	1.00
National free quitline?	**29** (29)	**30** (30)	1	1.00
Specialized cessation services covering the whole country or in selected areas?	**69** (68)	**69** (68)	0	1.00

The total base is 99—the number of countries who completed the survey in 2012 and 2015. The change is in percentage points. Bold data indicate percentages.

## Discussion

Our main finding is that implementation of FCTC Article 14 remains slow, with relatively little improvement during the last 3 years. Overall, in 2015, only three measures in our survey were reported to be in place in the majority of countries: 81% reported training some HCW to give brief advice, just more than half (54%) a nominated official responsible for tobacco dependence treatment and just more than 50% providing access to cessation support in primary care, pharmacies and hospitals. All other infrastructure and support systems were provided in only a minority of countries. Only 25% reported having an identified treatment budget, fewer than a third had an official treatment strategy (32%), fewer than half had official treatment guidelines (40%), only 30% mandated recording of tobacco use in medical notes, and there continued to be a clear income gradient in provision. For most measures, and with few exceptions, treatment provision was available in more high‐ than middle‐ and low‐income countries.

To our knowledge, this is the most detailed and comprehensive tobacco treatment survey conducted to date, sent to contacts in 94% of FCTC Parties at the time of the survey and achieving an 83% response rate. However, a survey of such scale has limitations. We used a purposive sample of contacts with expertise in tobacco cessation or tobacco control identified through various channels, some from national government, others outside government; hence, there is potential response bias and subjectivity in some responses. To minimize response bias, we encouraged the respondents to discuss and verify their responses with colleagues and, where possible, provide supporting information with their answers, such as copies of documents and links to websites. Wherever possible, we corroborated the responses using data from our previous survey or other sources. Differences in cultural context and respondents’ expertise could lead occasionally to differences in interpretations of questions about national cessation strategy and guidelines, or mass media campaigns*.* In particular, respondents may not distinguish between occasional advertisements on television and a major, comprehensive mass media campaign. When necessary, we corresponded with the respondents to check and clarify their answers.

Our main finding, that progress in Article 14 implementation is slow, is consistent with that of the 2014 WHO Global Progress Report 6. Based on 130 Parties’ implementation reports, the report estimated that the average implementation rate of the provisions under Article 14 was 51%, below that of other substantive articles: 84% for Article 8 (Protection from exposure to tobacco smoke); 70% for Article 11 (Packaging and labelling of tobacco products); 63% for Article 13 (Tobacco advertising, promotion and sponsorship); and 62% for Article 6 (Price and tax measures to reduce the demand for tobacco) 6.

As with the previous survey, we found a positive relationship between income level and treatment 4. This is not surprising, as many HIC had already had treatment infrastructure in place 7, 8, 9 prior to the FCTC entry into force in 2005, while for many middle‐ and low‐income countries, engagement with tobacco treatment might not have started before the adoption of the Article 14 implementation guidelines in 2010 3. If this trend in tobacco treatment provision across countries’ income level continues, it will lead inevitably to widening health inequality between HIC and middle‐ and low‐income countries.

The clear income gradient in almost all our main measures suggests that cost is (or is perceived to be) a barrier to the implementation of Article 14, with just a quarter of countries in the whole sample, and no LIC, having a clearly identified budget for tobacco dependence treatment. The Article 14 guidelines emphasize the importance of securing sustainable funding for cessation through tobacco price and tax increases, but clearly few countries have done so yet, although some countries (for example Panama, Korea) have identified adequate funding for treatment.

However, the Article 14 guidelines also stress the importance of using existing infrastructure and resources as much as possible, in order to keep costs down and facilitate quick action, and highlight several measures that are low cost. These include identifying tobacco users in medical notes and integrating brief advice by an HCW into the existing health‐care system, as well as developing an official national strategy and guidelines.

Recording tobacco use in all medical notes is essential to identify tobacco users and thus put the issue onto the health‐care system agenda, as well as providing an opportunity for brief advice to stop smoking. Brief advice given by a primary care physician has been shown to encourage quit attempts and can potentially be one of the most cost‐effective interventions when implemented throughout the health‐care system 7. According to a survey of smokers in 15 countries, between 50 and 70% of smokers visited a physician or other health‐care providers during 1 year 10. Our finding, that only 30% of countries record tobacco use in medical notes and only 44% provide brief advice as part of the existing health‐care services, demonstrates the potential for quick improvement in the provision of low‐cost broad‐reach interventions that would save many lives.

Delivering anti‐smoking messages and encouraging quit attempts via mass communication channels are measures recommended by both Article 14 and Article 12 guidelines 3, 11. Mass media campaigns in particular can potentially have wide population reach and low per‐capita cost 12. However, such campaigns require substantial funding and may not be easily affordable in middle‐ and low‐income countries. Our findings showed that just more than half of respondents reported that their countries ran mass media campaigns promoting cessation, and this increased by only one percentage point during the last 3 years. Effective mass media campaigns 13 can be adapted for use with smokers in different countries, thus reducing the set‐up and production cost substantially. Self‐help materials (leaflets, booklets), printed or distributed electronically, is another affordable measure 14 that can be effective in lower‐income countries with widespread literacy and internet access. However, evidence suggests that adequately funded mass media campaigns need to be sustained over a period of time to be optimally effective as part of a comprehensive tobacco control strategy 15, 16.

We found that free national quitlines were available in only 23% of countries (Table 2), and increased by just one percentage point during the last 3 years in our cohort of 99 countries (Table 5). Quitlines require investment in infrastructure, people and training. As demand for cessation continues to grow in middle‐ and low‐income countries 17, 18, text messaging might be a cheaper alternative to quitlines. There is emerging evidence of the effectiveness of behavioural support delivered via automated mobile phone text messaging 14.

Although medicines availability is excellent in HIC (for example almost all HIC have NRT; Table 3), the availability of affordable cessation medications remains an issue for middle‐ and low‐income countries, including those medications considered to be globally affordable by a WHO definition 14. We found that nicotine replacement therapy (NRT) patches and gum, which have been on the WHO List of Essential Medications since 2009 19, 20, are available in just more than half of LMIC and approximately half of LIC, and their affordability is low. Cytisine, a low‐cost medication, estimated to be 25 times cheaper than varenicline 21, is not licensed as a cessation medication outside countries in Central and Eastern Europe, and was available in fewer than 20% of middle‐ and low‐income countries.

Cessation medications have been shown to increase the success rate of cessation attempts 22, 23. The Article 14 guidelines recommend that countries should make cessation medications available for tobacco users wanting to quit, and where possible provide them free or at an affordable cost. As cigarettes are becoming more affordable over time in middle‐ and low‐income countries 24, countries need to implement measures to reduce the cost of NRT and license low‐cost cessation medications such as cytisine, estimated to be a globally affordable intervention 14 .

Our cohort data showed very little change in specialized treatment services. Although all countries should aim eventually for comprehensive treatment provision, free nation‐wide specialized cessation services such as those in England clearly require a national cessation strategy and government funding 25, and thus are unlikely to be feasible in middle‐ and low‐income countries. An additional consideration here is coverage, as they tend to reach a small proportion of the population. Were such specialized support to be delivered by trained multiple providers, including GPs, nurses, dentists, pharmacists and community workers, as well as cessation specialists, and also delivered through telephone counselling 26, 27, then they might achieve better reach. However, we believe middle‐ and low‐income countries should follow the Article 14 guidelines by first prioritizing core infrastructure broad‐reach low‐cost measures.

The main recommendations of Article 14 and its guidelines were that countries should develop and disseminate national treatment guidelines and a national cessation strategy, yet this has been achieved by only 40% of countries, an increase of only 9 percentage points in the last 3 years.

FCTC A14 implementation is slow and shows a significant gradient by income level, with less treatment provision the lower the income. If the current pattern of implementation continues the gap between HIC and middle‐ and low‐income countries will grow wider, worsening existing health inequalities between countries. Implementation needs to be improved, with an emphasis on affordable, broad‐reach measures. This means prioritizing core infrastructure measures, including developing an official national cessation strategy and guidelines, mandatory recording of tobacco use in medical notes and helping HCW to stop using tobacco, along with broad‐reach low‐cost interventions, including brief advice and text messaging.

## Declaration of interests

None.

## Supporting information


**Appendix S1** Tobacco treatment questionnaire.
