# Supplementary material for: Progress in implementation of WHO FCTC Article 14 and its guidelines: a survey of tobacco dependence treatment provision in 142 countries
Source: Addiction. 2017 Aug 2;112(11):2023–31. doi: 10.1111/add.13903 (PMC5655744; doi:10.1111/add.13903)
Supplement: Supplementary file 1 — Appendix S1 Tobacco treatment questionnaire. [file ADD-112-2023-s001.doc]

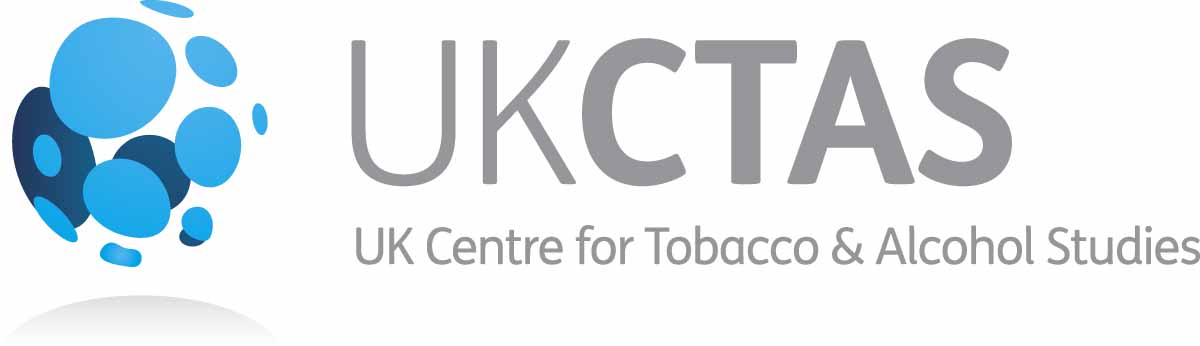


| **Tobacco Treatment Survey 2015** |
| --- |

| Thank you very much for helping us with this survey. We think the questionnaire should take approximately 15 minutes. If you have any questions about the survey please email us.  Martin Raw [martin@martinraw.com](mailto:martin@martinraw.com) and Kapka Nilan [msxkn4@nottingham.ac.uk](mailto:msxkn4@nottingham.ac.uk) on behalf of the survey team at Nottingham University (Rachael Murray) and King’s College London (Ann McNeill). |
| --- |

| Definitions of terms used in this survey can be found in the FCTC Article 14 guidelines:  <http://www.who.int/fctc/Guidelines.pdf?ua=1>  Under each question we have left space for comments if you wish to add any additional information or clarify your responses. |
| --- |

| **1) Contact information** |
| --- |
| Name: |
| Position: |
| Organisation: |
| Country: |
| Email: |

| **2) Is there an officially identified person in government (or contracted by government) who is responsible for tobacco dependence treatment?** | |
| --- | --- |
| Yes |  |
| No |  |
|  | |

| **3) Does your country have a clearly identified budget for tobacco dependence treatment?** | |
| --- | --- |
| Yes |  |
| No |  |
|  | |

| **4) Does your country have an official, written, national tobacco treatment strategy?** | |
| --- | --- |
| Yes |  |
| No |  |
| If yes please give a link and/or PDF if possible. | |

| **5) Does your country have official national tobacco treatment guidelines?** | |
| --- | --- |
| Yes |  |
| No |  |
| If yes, approximately what year were they published? | |
| If yes please give a link and/or PDF if possible. | |

| **6) Does your country run mass media campaigns promoting cessation?** | |
| --- | --- |
| Yes |  |
| No |  |
| If yes, please provide the year of the most recent campaign if known. |  |
|  | |

| **7) Does your country offer help to healthcare workers and other relevant groups to stop using tobacco?** | |
| --- | --- |
| Yes |  |
| No |  |
|  | |

| **8) Is it mandatory to record patients’ tobacco use in medical notes in your country?** | |
| --- | --- |
| Yes |  |
| No |  |
|  | |

| **9) Is brief advice integrated into existing services such as (but not limited to) primary care, tuberculosis, HIV/AIDs, etc?** | |
| --- | --- |
| Yes |  |
| No |  |
| If yes, could you give some details, for example, which services. | |

| **10) Are healthcare workers trained to give brief advice?** | |
| --- | --- |
| Yes all |  |
| Yes some |  |
| No |  |
| If yes, could you give some details, for example which groups are trained. | |

| **11) Is tobacco cessation incorporated into the training curricula of healthcare students and workers?** | |
| --- | --- |
| Yes all |  |
| Yes some |  |
| No |  |
| If yes, could you give some details, for example, which groups are trained. | |

| **12) Does your country have a national telephone tobacco quitline, free to callers?** | |
| --- | --- |
| Yes, free national quitline or quitlines in all major regions of the country |  |
| Yes, free national quitline but not tobacco only, it is part of a quitline that includes other issues |  |
| Yes, free quitlines but only in selected regions of the country |  |
| No |  |
|  | |

| **If no please go to Q14** |
| --- |

| **13) If you answered 'yes' to any of the telephone quitline questions** | | |
| --- | --- | --- |
|  | Yes | No |
| Does it have people answering always or almost always? |  |  |
| Does it offer multiple sessions with counsellors calling back to offer support? |  |  |
| Does it refer callers to local specialist treatment services? |  |  |
| Does it offer information about tobacco cessation medications? |  |  |
| Is the quitline number on tobacco packaging? |  |  |
|  | | |

| **14) Does your country offer a text messaging service or services to tobacco users?** | |
| --- | --- |
| Yes |  |
| No |  |
|  | |

| **15) Does your country have specialised tobacco dependence treatment services (experts or units/clinics) offering individual or group support delivered by trained professionals?** | |
| --- | --- |
| Yes, a network of treatment support covering the whole country |  |
| Yes, treatment support but only in selected areas |  |
| No |  |
|  | |

| **If no please go to Q18; If yes please answer Q16 and Q17** |
| --- |

| **16) Are these services free to users?** | |
| --- | --- |
| Yes |  |
| Partially |  |
| No |  |
| We realise the answer to this question may be complex; please give as much detail as possible. | |

| **17) Does your country monitor the specialised treatment services (including numbers using the services and outcome)?** | |
| --- | --- |
| Yes |  |
| No |  |
|  | |

| **18) Are the following medications available in your country?** | | |
| --- | --- | --- |
|  | Yes | No |
| NRT gum |  |  |
| NRT patch |  |  |
| Any other NRT |  |  |
| Bupropion |  |  |
| Varenicline |  |  |
| Nortriptyline |  |  |
| Cytisine (Tabex) |  |  |
| Other (please give details): | | |
|  | | |

| **19) Are the following medications easily affordable to most tobacco users in your country?** | | |
| --- | --- | --- |
|  | Yes | No |
| NRT gum |  |  |
| NRT patch |  |  |
| Any other NRT |  |  |
| Bupropion |  |  |
| Varenicline |  |  |
| Nortriptyline |  |  |
| Cytisine (Tabex) |  |  |
|  | | |

| **20) In general is NRT more or less expensive than smoking in your country?** | |
| --- | --- |
| More expensive |  |
| Less expensive |  |
| More or less the same |  |
| Don’t know |  |
|  | |

| **21) Can tobacco users get help to stop smoking in the following settings?** | | |
| --- | --- | --- |
|  | Yes | No |
| General/family practice |  |  |
| Pharmacists |  |  |
| Dentists |  |  |
| Hospitals |  |  |
| Addiction services |  |  |
| Work places |  |  |
| Educational institutions |  |  |
| Mental health units |  |  |
| Prisons |  |  |
| Traditional health practitioners / healers |  |  |
| Please add any other relevant groups/settings: | | |

| **22) Are electronic cigarettes available in your country?** | | |
| --- | --- | --- |
|  | Yes | No |
| Through the internet |  |  |
| In ordinary shops |  |  |
| Over-the-counter in pharmacies |  |  |
| As a licensed medication for tobacco dependence treatment |  |  |
|  | | |

| **23) Are the following tobacco products widely used in your country?** | | |
| --- | --- | --- |
|  | Yes | No |
| Cigarettes |  |  |
| Water pipes (shisha, narghile, hookah) |  |  |
| Smokeless tobacco (nasal, oral) |  |  |
| Other (please name and/or describe) | | |
|  | | |

| **24) What in your opinion are the main barriers or challenges to developing further tobacco dependence treatment in your country?** |
| --- |
|  |

| **25) Would you be willing to be interviewed by us about tobacco dependence treatment in your country?** | |
| --- | --- |
| Yes |  |
| No |  |

| **26) Please add here any further comments you would like to make, or data, including any relevant documents and references.** |
| --- |
|  |

| **Survey complete; thank you very much indeed.** |
| --- |
